# A rapid multi-parametric quantitative MR imaging method to assess Parkinson’s disease: a feasibility study

**DOI:** 10.1186/s12880-024-01229-0

**Published:** 2024-03-05

**Authors:** Min Duan, Rongrong Pan, Qing Gao, Xinying Wu, Hai Lin, Jianmin Yuan, Yamei Zhang, Lindong Liu, Youyong Tian, Tong Fu

**Affiliations:** 1 Department of Radiology, Jiangsu Province Hospital of Chinese Medicine, Affiliated Hospital of Nanjing University of Chinese Medicine, Nanjing, China; 2 Department of Neurology, Nanjing First Hospital, Nanjing Medical University, No.68, Changle Road, 210006 Nanjing, Jiangsu Province China; 3 Department of Radiology, Nanjing First Hospital, Nanjing Medical University, No.68, Changle Road, 210006 Nanjing, Jiangsu Province China; 4 Central Research Institute, United Imaging Healthcare, Shanghai, China

**Keywords:** Quantitative imaging, Parkinson’s disease, Subcortical nuclei, Dopaminergic midbrain nuclei, Multi-parametric MRI

## Abstract

**Background:**

MULTIPLEX is a single-scan three-dimensional multi-parametric MRI technique that provides 1 mm isotropic T1-, T2*-, proton density- and susceptibility-weighted images and the corresponding quantitative maps. This study aimed to investigate its feasibility of clinical application in Parkinson’s disease (PD).

**Methods:**

27 PD patients and 23 healthy control (HC) were recruited and underwent a MULTIPLEX scanning. All image reconstruction and processing were automatically performed with in-house C + + programs on the Automatic Differentiation using Expression Template platform. According to the HybraPD atlas consisting of 12 human brain subcortical nuclei, the region-of-interest (ROI) based analysis was conducted to extract quantitative parameters, then identify PD-related abnormalities from the T1, T2* and proton density maps and quantitative susceptibility mapping (QSM), by comparing patients and HCs.

**Results:**

The ROI-based analysis revealed significantly decreased mean T1 values in substantia nigra pars compacta and habenular nuclei, mean T2* value in subthalamic nucleus and increased mean QSM value in subthalamic nucleus in PD patients, compared to HCs (all p values < 0.05 after FDR correction). The receiver operating characteristic analysis showed all these four quantitative parameters significantly contributed to PD diagnosis (all p values < 0.01 after FDR correction). Furthermore, the two quantitative parameters in subthalamic nucleus showed hemicerebral differences in regard to the clinically dominant side among PD patients.

**Conclusions:**

MULTIPLEX might be feasible for clinical application to assist in PD diagnosis and provide possible pathological information of PD patients’ subcortical nucleus and dopaminergic midbrain regions.

## Background

Parkinson’s disease (PD) is one of the most prominent neurodegenerative disorders. It is characterized by a progressive loss of dopaminergic neurons, which accumulated in the substantia nigra pars compacta(SNpc) [1]. Dopamine within dopaminergic neurons undergoes various processes, encompassing synthesis, storage, release, reuptake, and degradation pathways [2]. The decreased dopamine concentration in PD patients leads to symptoms manifesting as tremors, rigidity, bradykinesia, and postural instability [3]. Recent studies have suggested PD-related dopaminergic system dysfunction evolved deep gray matter nucleus beyond the SNpc [4, 5]. The alterations of the subcortical nucleus and midbrain nucleus contribute to the fluctuation of dopamine, including dopamine neuron-rich regions (e.g., SNpc), midbrain nucleus that activate or inhibit dopaminergic neurons (e.g., thalamus), and subcortical nucleus that are modulated by dopamine release (e.g., caudate nucleus) [2]. However, quantitative detection of the microstructural alterations of the subcortical nucleus and midbrain nucleus in vivo is still challenging.

Magnetic resonance imaging (MRI) is an important tool to observe the brain in vivo and has been applied in clinics for the diagnosis and monitoring of various neurological disorders. However, PD patients present no specific sign in clinic routine brain MRI scans. Sophisticated MRI techniques providing specific imaging features related to PD pathology with higher sensitivity for early PD diagnosis are desirable. Measurements of quantitative MRI (qMRI), such as T1, T2 and T2* relaxation times, proton density and quantitative susceptibility mapping (QSM), offer valuable insights into the microstructural changes associated with PD [7–13]. QSM value variations were reported in deep grey matter nucleus in early PD patients to be associated with regional iron deposition, especially the increased QSM values found in substantia nigra (SN) and red nucleus (RN) contralateral to most affected limb in early PD [10]. T2* parameter alterations in SN were also considered to be associated with iron deposition in PD progress [8, 13]. Reduction of proton density associated with the macromolecular free water content decrease were reported in dopamine-innervated gray matter regions in early PD [9]. T1 relaxation time alterations were detected in SN, RN, subcortical gray matter ar//eas and motor cortex areas in early PD, which were possibly associated with neuronal loss, meso-cortical dopaminergic innervation loss and unmyelinated dopaminergic axons degeneration that correspond to PD pathology and motor dysfunction [7–9]. It is argued that T2 relaxation time alteration in the contralateral mesencephalon might be associated with dopaminergic neuron death and gliosis [9]. Radiomics approach captured texture changes of T2 weighted image in SN that may correlate to microstructural alteration in nigrostriatal degeneration in PD [11]. However, to optimal brain MRI scans’ contribution to PD diagnosis and clinical evaluation, all these important abnormalities better be detected and evaluated simultaneously.

Multiple quantitative MR imaging parameters providing comprehensive information of subtle brain tissue alterations have potential benefits in PD diagnosis and expand current persecutive in the pathological progress of PD. However, qMRI measurements of proton density, T2*, and QSM are not yet routine in clinical practice due to longer scan times. This prolonged stillness during MRI scanning poses challenges for PD patients with tremors and bradykinesia. Multiple quantitative MR parameters in one single scan with a shorter scan time would reduce patients’ discomfort and the diminish possibility of misregistration of relevant anatomic information among different imaging sequences obtained at different times.

MULTIPLEX is a single-scan 3D multi-parametric MR imaging method, providing 1 mm isotropic T1-, T2*-, proton density- and susceptibility-weighted images, and also the corresponding quantitative maps with the scanning time of around 10 min [14]. Compared to previous brain multiparametric quantitative MRI methods, MULTIPLEX showed advantages in the improvement of signal-to-noise ratio (SNR) and flexibility [14, 15]. In this study, quantitative parameters of proton density, T1, T2*, and QSM derived from MULTIPLEX were measured and analyzed in the subcortical nucleus and midbrain regions of PD. We aimed to bridge the gap between advanced MRI technology and its clinical application in PD.

## Methods

### Participants and clinical examinations

This study was conducted with the ethical approval of the local Ethics Committee of Nanjing first hospital (Number KY20220701-04). All participants gave their written informed consent. Patients were recruited from the neurological wards, the outpatient clinic, and local patient support groups. The diagnosis of PD was conducted according to the Movement Disorder Society (MDS) diagnostic criteria for PD by a movement disorder specialist [16]. The Hoehn and Yahr (H&Y) scale was used to classify the disease phase, non-dementia PD patients with H&Y scale from I to III were recruited [17]. Patients with atypical Parkinsonism and other known brain pathologies (e.g., stroke, small vessel disease, or tumor) were excluded. Patients with clinical conditions interfering with MRI diagnostics, like severe head tremor, dystonia, or dyskinesia, had to be excluded as well. PD patients with severe comorbidities were also excluded. PD patients’ information such as education years, disease duration, and clinically dominant side were recorded. Symptoms of PD patients were assessed by the Movement Disorder Society sponsored revision of the Unified Parkinson’s Disease Rating Scale (MDS-UPDRS scores) [18]. Patients were rated in the best medication “on” state. Healthy subjects recruited from the local population were also reviewed as healthy controls (HCs), who matched to patients in respect to age and sex. Subjects with neurological diseases, other major systemic disorders, cognitive or psychiatric impairments were excluded according to the self-report. Participants with abnormal MR findings like intracranial mass, ischemia sign, or brain hemorrhage were also excluded. All participants were right-handers according to the self-report.

### MR examinations and data processing

The MR scanning, using the MULTIPLEX method, was performed on a 3.0 Tesla system (uMR 780, United Imaging Healthcare, Shanghai, China) using a 24-channel head-neck coil. The MULTIPLEX was performed with the following parameters: dual flip angle α1/α2 = 4°/16°, dual repetition time (TR)1/TR2 = 9.8/28.8 ms, seven echoes with echo time (TE) = 7.4$\sim$22.1 ms and ΔTE = 4.9 ms (bipolar readouts), bandwidth = 380 Hz/px, matrix size = 240 × 240 × 176, voxel size = 1 × 1 × 1 mm3, and scanning time of 10:15. The reconstruction and processing of MULTIPLEX images were automatically performed using in-house C + + programs on the Automatic Differentiation using Expression Template platform (United Imaging Healthcare, Shanghai, China) platform.

The MULTIPLEX sequence produced T1, T2*, proton density, and susceptibility-weighted images and also the corresponding quantitative maps without any extra scan time. The reconstructed T1, T2*, proton density and QSM maps were all in the same spatial coordinate. The T1 map was non-linearly registered to the standard Montreal Neurological Institute coordinate space (the ICBM152 template), using the Advanced Normalization Tools. The HybraPD atlas provides a fine parcellation of subcortical nuclei with accurate external boundary definitions for PD patients [6] In the standard space, it consists of 12 pairs human brain subcortical nuclei: putamen (Pu), caudate nucleus (CN), nucleus accumbens (NAC), ventral pallidum (VeP), internal and external globus pallidus (GPi and GPe), pars reticulata and pars compacta of substantia nigra (SNr and SNc), red nucleus (RN), subthalamic nucleus (STN), habenular nuclei (HN), and thalamus (Thal). Through the inverse non-linear transformation, all the subcortical nuclei were located in the subject’s space on corresponding T1, T2*, proton density, and QSM images.

**Fig. 1:**
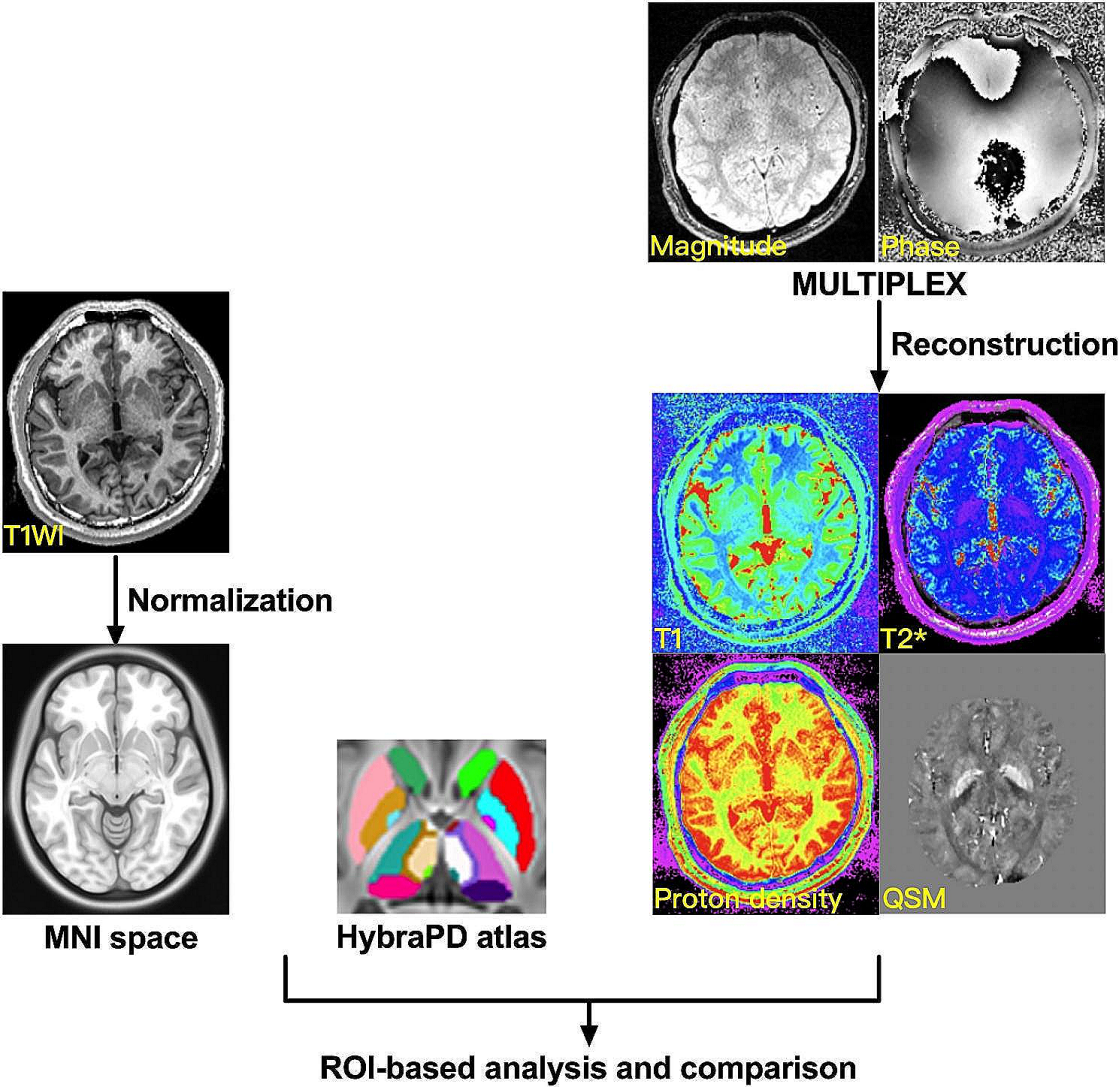
The flowchart of data processing. After image reconstruction, MULTIPLEX produces four kinds of quantitative parameters: T1, T2*, proton density, and QSM. The T1 map was non-linearly registered to the standard Montreal Neurological Institute coordinate space. Through the inverse non-linear transformation, all the subcortical nuclei were located in the subject’s space on corresponding T1, T2*, proton density, and QSM images according to the HybraPD atlas consisting of 12 human brain subcortical nuclei. The mean values of these four quantitative parameters were extracted from each subcortical nucleus. Afterward, ROI-based analyses were applied. HC: healthy control, PD: Parkinson’s disease, QSM: quantitative susceptibility mapping, T1WI: T1-weighted image, MNI: Montreal Neurological Institute

Mean values representing the averaged parameter of corresponding quantitative maps across all voxels (3D pixels) within each ROI were extracted from quantitative maps of T1 relaxation time, T2* relaxation time, proton density and QSM for each subcortical nucleus, using Matlab (MathWorks, Natick, MA). Thus, a total of 48 parameters (averaged values of two hemispheres) that represented the quantitative properties of potential PD-related regions were extracted from each participant’s MULTIPLEX data. The flowchart of data processing was exhibited in Fig. 1.

### Statistical analysis

The Kolmogorov-Smirnov tests were applied to check the normality of all the MR imaging quantitative parameters. If they were normally distributed, the t-tests were used for group comparison. If not, the Mann-Whitney tests were conducted, instead. A statistical threshold was set at *p* < 0.05 after false discovery rate (FDR) correction for multiple comparisons. FDR multiple comparisons correction was performed by using the script of mafdr in Matlab with the default parameters. The diagnosis ability of possible results derived from the four quantitative maps was further evaluated using a receiver operating characteristic (ROC) curve. The Spearman’s correlation analysis was conducted between each quantitative parameter with significant group difference and the MDS-UPDRS III score. The Wilcoxon matched-pairs signed rank test was also performed for each quantitative parameter with significant group difference between contralateral and ipsilateral hemispheres in regard to the clinically dominant side among PD patients. Subsequently, the power analysis was performed using the scripts of sampsizepwr and binofit in Matlab (MathWorks, Natick, MA) to calculate the power for the sample size in this study. The power analysis was based on the significant group differences and associations of quantitative parameters with PD and motor symptom severity.

## Results

### Demographic and clinical characteristics of subjects

The demographic characteristics and clinical assessment of the PD patients and the HCs were summarized in Table 1. It showed no notable discrepancies in age and gender between PD patients and HC, performing a Fisher’s test for gender and a t-test for age. Twenty-seven PD patients (age: 68.9 ± 9.2 years, disease duration: 3.6 ± 2.9 years, 17 females) and twenty-three healthy subjects (mean = 65.9 ± 7.2 years old, 15 females) were included in this study. Motor deficits of our PD subjects in the MDS-UPDRS part III scored a mean of 30.7 points (standard deviation of 13.2) pointing to mild disease severity [19]. Our PD patients showed the H&Y stage at 2.1 ± 0.7. In our PD group, fourteen patients manifested clinically dominant side on the left, and thirteen on the right.

**Table 1:** The demographic and clinical outcome of all participants

	PD Patients (*n* = 27)	Healthy controls (*n* = 23)	p-value
Age (years)	68.9 ± 9.2	65.9 ± 7.2	0.21^a^
Gender (male/female)	10/17	8/15	0.87^b^
Disease duration (years)	3.6 ± 2.9	NA	NA
Clinically dominant side (left/right)	14/13	NA	NA
H&Y stage	2.1 ± 0.7	NA	NA
MDS-UPDRS part I	10.8 ± 8.7	NA	NA
MDS-UPDRS part II	12.2 ± 7.3	NA	NA
MDS-UPDRS part III	30.7 ± 13.2	NA	NA
MDS-UPDRS part IV	0.9 ± 2.3	NA	NA

H&Y stage, Hoehn and Yahr stage; MDS-UPDRS, Movement Disorders Society Unified Parkinson’s Disease Rating Scale; NA, not applicable

Values are represented as the mean ± standard deviation, except for the gender distribution

^a^The p value was calculated with a t-test

^b^The p value was obtained using a Fisher’s test

### ROI-based quantitative MR parameters analysis

**Fig. 2:**
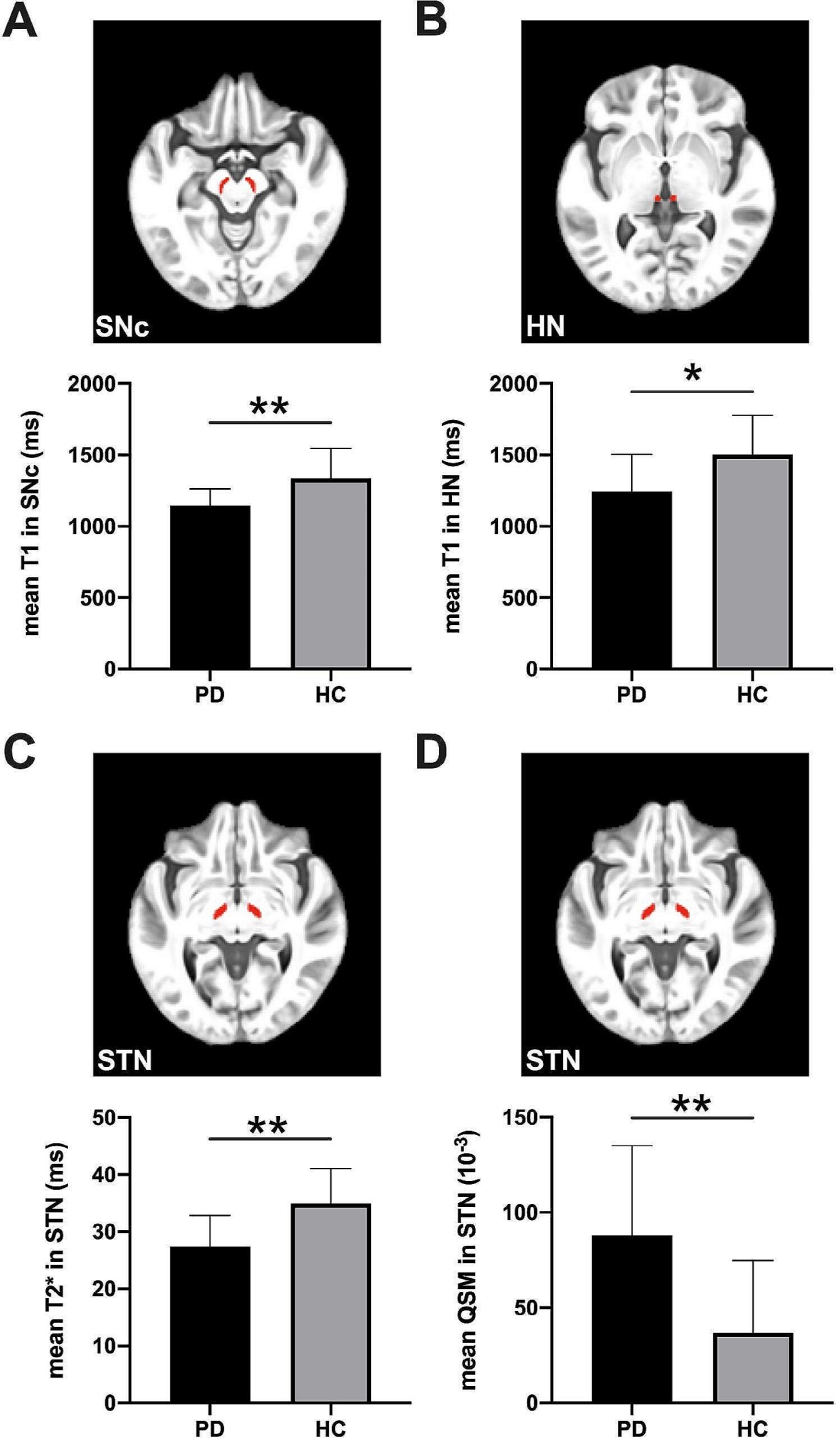
The comparisons of quantitative parameters between Parkinson’s disease (PD) patients and healthy control (HC). PD patients showed (**A**) decreased mean T1 value in substantia nigra pars compacta (SNc), (**B**) decreased mean T1 value in habenular nuclei (HN), (**C**) decreased mean T2* value in subthalamic nucleus (STN) and (**D**) increased mean QSM value in subthalamic nucleus(STN). Statistical significance is indicated by asterisks (*, *p* < 0.05; **, *p* < 0.01 after FDR correction). HC: healthy control, PD: Parkinson’s disease

Kolmogorov-Smirnov tests showed that the imaging quantitative measurements obtained were not normally distributed. Compared to HC, PD patients showed decreased mean T1 values in SNc, decreased mean T1 values in HN, decreased mean T2* values in STN and increased mean QSM values in STN (all p values < 0.05, after FDR correction; Fig. 2). No significant difference in mean proton density values between PD and HC was found. The diagnostic performance of these four quantitative parameters derived from ROC analysis showed that mean T2* value in STN has the optimal performance with area under the curve (AUC) value at 0.83 (p-value < 0.001), following mean QSM value in STN, mean T1 value in SNc, mean T1 values in HN, with AUC values of 0.80 (p-value = 0.001), 0.78 (p-value = 0.002), and 0.750 (p-value = 0.006), respectively (Table 2).

**Table 2:** Diagnostic performance of the quantitative parameters with significant differences

Parameters	Cut-off value	AUC	Sensitivity (%)	Specificity (%)	p-value
mean T1 value in SNc	< 1180	0.78	66.7	78.3	0.002
mean T1 value in HN	< 1184	0.75	55.6	87.0	0.006
mean T2* value in STN	< 29.8	0.83	77.8	78.3	< 0.001
mean QSM value in STN	> 0.051	0.80	77.8	69.6	0.001

AUC: area under the curve; HN: habenular nuclei; QSM: quantitative susceptibility mapping; SNc: substantia nigra pars compacta; STN: subthalamic nucleus

**Fig. 3:**
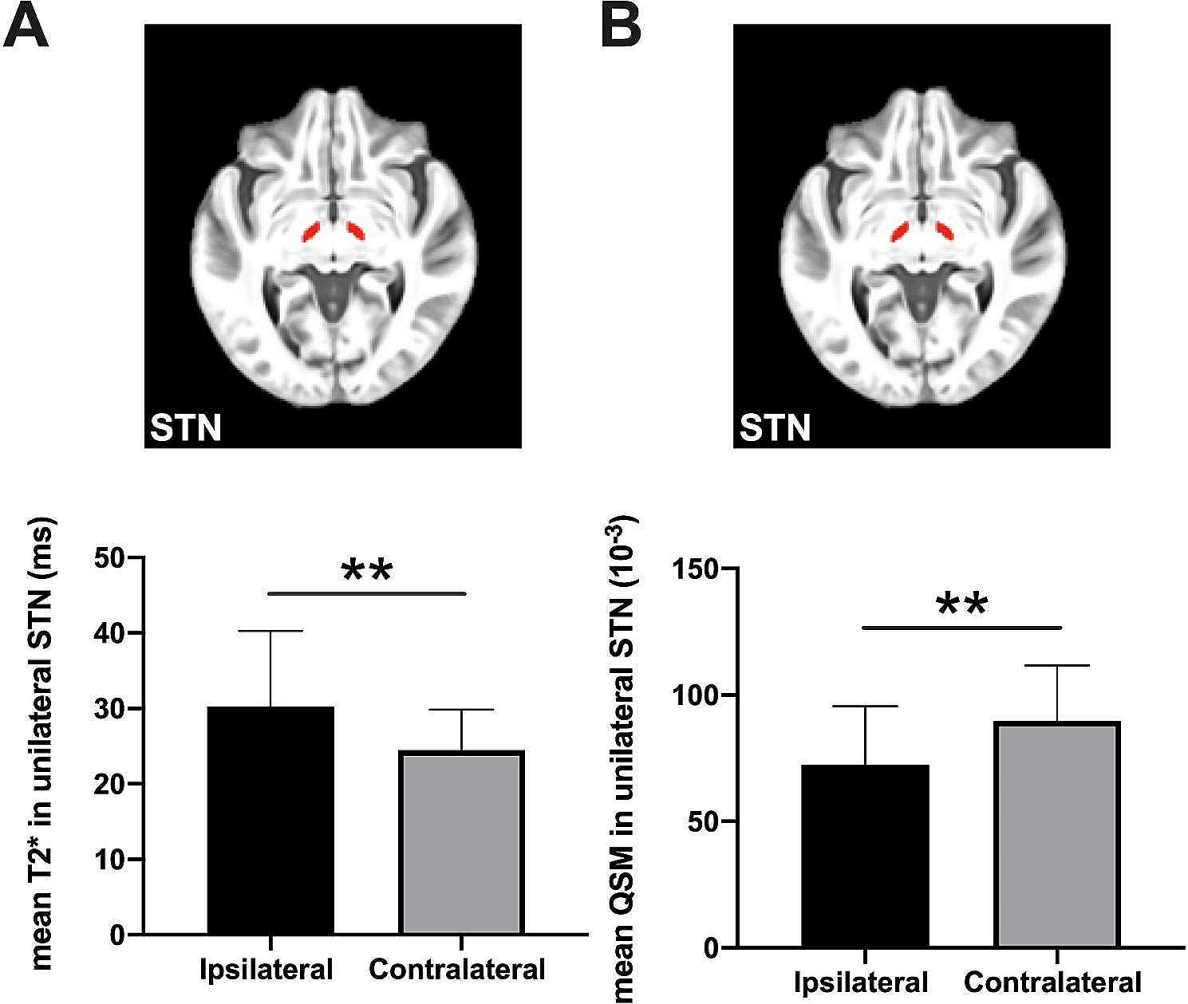
The paired comparisons of quantitative parameters between two hemispheres in Parkinson’s disease(PD) patients. The mean T2* value (**A**) and mean QSM value (**B**) in unilateral STN showed significant differences between contralateral and ipsilateral hemispheres in regard to the clinically dominant side among PD patients. Statistical significance is indicated by asterisks (**, *p* < 0.01 after FDR correction). HC: healthy control, PD: Parkinson’s disease

The mean T2* value in unilateral STN and mean QSM value in unilateral STN showed significant differences between contralateral and ipsilateral hemispheres regarding the clinically dominant side among PD patients, in details: The mean T2* value in contralateral STN was significantly lower than those in the ipsilateral hemisphere, plus the mean QSM value in contralateral STN was significantly higher than that in the ipsilateral hemisphere (all p values < 0.01, after FDR correction; Fig. 3).

No significant correlation was found between the MDS-UPDRS III score and the quantitative parameters with significant group differences in PD patients (all the absolute values of *r* < 0.25; all p values > 0.05, after FDR correction).

### The result of power analysis

Based on the current sample size of 27 patients and 23 healthy subjects, the powers of the significant differences of quantitative parameters between PD and HC groups, and also between contralateral and ipsilateral hemispheres in PD patients were all larger than 0.70.

## Discussion

In this study, we applied the MULTIPLEX method to obtain multi-parametric qMRI measurements of subcortical nuclei in PD patients. In our results, mean T1 values in SNc and HN, mean T2* value in STN and mean QSM value in STN showed significant differences between PD and HC. These four parameters could significantly contribute to PD diagnosis with AUC values ranging from 0.75 to 0.83. In consideration of the asymmetric motor symptom characteristic in PD, alterations of mean T2* value and mean QSM value in unilateral STN showed symptom-related lateralization in PD patients.

This study conducted a data-driven analysis of multiple quantitative MRI parameters for PD diagnosis. It characterized probable PD pathobiological changes. By integrating various MR measurements, including those reflecting dopaminergic neuron loss, α-synuclein aggregation, and iron deposition-induced neuroinflammation, multiple qMRI researches have been conducted [1, 9, 11, 20, 21]. Data-driven methods would effectively maximize the amount of complementary information derived from multiple quantitative MRI measurements [20, 22] Moreover, we segmented the subcortical nuclei by applying the HybraPD atlas, which was generated by using fused multimodal MR images and provided manually delineated 12 pairs of bilateral subcortical nuclei that are highly related to PD pathology [6]. The MULTIPLEX method acquired multi-contrast information in the PD-specifical parcellated subcortical structures parallelly showed advantages in detecting early alterations of brain microstructure and in better understanding the pathology heterogeneity of PD.

In our results, T1 maps showed sensitivity in detecting brain microstructure alteration in PD, which showed consistency with previous studies [9, 23]. The alteration of T1 relaxation time has been considered to represent water molecule changes in brain microstructure and also may be associated with dopaminergic neuron loss and axon demyelinationn [9, 13, 15, 24].The significant decrease in mean T1 values in SNc in our results aligns with PD pathology, indicating potential diagnostic value [2]. However, in our results, the SNr did not show any alteration that contributes to PD diagnosis. This may be because SNr mainly conveys signals from basal ganglia to other brain areas and changes after SNc [25]. Habenula nuclei is an ancient brain structure that controls the dopaminergic system through direct or indirect interventions to regulate various motivational cognitive and motor processes [26, 27]. The significantly decreased mean T1 value in HN in PD patients may correspond to the early microstructural alteration of HN in Parkinson’s disease pathology progress. Moreover, T1 signal alterations were reported to be related to microglia activation, which plays an important role in neuroinflammation [28, 29]. Thus, the alterated T1 mean values in above structures potentially linked to neuroinflammation, a critical component in PD pathogenesis.

Our quantitative MR imaging results may provide important information for clinicians in diagnosis prognosis and possible treatment planning for PD patients. In our results, the mean T2* in the subthalamic nucleus (STN) and mean QSM in STN showed the most obvious differences between PD patients and HCs (with AUC values at 0.83 and 0.80, respectively). Both parameters of STN contribute to differentiating PD from HC independently (Fig. 2, C and D), and also showed the lateralization feature in PD (Fig. 3). Both the T2* relaxometry contrast alteration and signal alteration of QSM are considered to be associated with abnormal iron deposition, which is reported as the pathologic cause of PD and a possible imaging marker for PD diagnosis [8, 10, 32]. STN is an important component in the pathology path of PD, it becomes hyperactive to enhance dopamine generation when the dopaminergic deficit exceeds a given threshold (around 60%) in the prodromal motor period of PD [30, 31]. Subsequently, abnormal neuronal activities in STN would then lead to the deficit of the motor circuit, which manifests as unilateral motor features [31]. Meanwhile, the decreased mean T2* value in contralateral STN and increased mean QSM in contralateral STN in PD patients representing the iron deposition of STN in the contralateral hemisphere were more severe than those in the ipsilateral hemisphere. The asymmetric brain alteration is in line with the PD pathological pattern that subcortical gray matter degenerates more severely in the hemisphere contralateral to the onset-side reported by a previous study [33]. As the major deep brain stimulation (DBS) therapy target structure, STN showed lateralization feature in our PD patients which may be the prognosis reference of motor symptom improvement after DBS [30, 34].

Compared to previous studies, our results showed some heterogeneity [15, 24, 35–37]. Previous results demonstrated no significant QSM alteration was found in early-stage PD, which has been attributed to the time-specific characteristics of iron deposition in PD [35] The heterogeneous results may be because of the heterogeneity of PD pathological paths and the possible compensatory processes in the brain subcortical nuclei and midbrain nuclei [36, 37]. Also, we found no significant difference of proton density maps in brain subcortical nuclei between our PD and HC groups. It may be because proton density majorly indicates an alteration of free water content in brain tissue rather than water molecules in neurons [15, 24]. Meanwhile, the proton density map would be affected by the pulse sequence weighting factor and the receiver coil sensitivity profile, which may lead to inaccuracies depending on the scanner control variables and homogeneity in the receiver coil sensitivity profile [38].

Our results derived from the MULTIPLEX method have the potential to provide clinically valuable information. The outcome of imaging features derived from MULTIPLEX multiple MR parameters including T1, T2*, proton density, and QSM in 12 pairs of subcortical nuclei which are dedicatedly defined according to HybraPD atlas showed possible capability in differentiating PD and HCs. There was no significant difference in age between our PD and HC group (Table 1), thus in our results, the effect of age had not been considered. No correlation between the quantitative MR assessments and the MDS-UPDRS III scores was found, which has been observed by previous studies [7, 9]. A possible reason could be that the brain tissue alteration in our PD group (at the early disease stage with an H&Y at 2.1 ± 0.7) might be negligible for the correlation analysis. It is noteworthy that obtaining multiple-contrast sequences requires a prolonged scanning time, which is not practical in the clinic routine. Similar to our study, Klietz et al. obtained mean values of multiple quantitative MRI parameters on specific structures on separately scanned images (proton density, T1 relaxation time, T2, and T2 prime) by manually drawing a certain-sized circle as regions of interest (ROIs) [9] This approach not only incurs a lot of labor and time and is impractical for clinical applications but also fails to provide information about the entire target structures. It is also noteworthy that radiomics had been applied to extract quantitative parameters to identify PD-related brain alterations [11, 12] however, the extracted radiomics results, like texture feature, joint entropy, zone entropy, etc., are derived from invisible mathematic models and are difficult to interpret in terms of their association to pathology and symptoms. Functional MR imaging (fMRI) and diffusion tensor imaging (DTI) are widely applied to capture PD-associated brain activity, functional and structural connectivity changes [39–41] However, the Blood-oxygenation-level-dependent (BOLD) signal variation derived from fMRI and the diffusion index results in different studies showed heterogeneity. Moreover, the approach presents difficulties in individual clinical applications. Obtaining mean values of multiple quantitative MRI parameters in predefined PD vulnerable regions in this study only provides information about microstructural brain alterations. Still, the MULTIPLEX method has the advantage of acquiring these parameters in one single scan in a relatively short time, which provides a chance to improve the clinical feasibility of applying multiple contrasts MR imaging in PD.

To make full use of the multi-parametric nature of data, the MULTIPLEX incorporated the recently proposed multi-dimensional integration (MDI) image processing strategy for the calculation of quantitative parameters [14] MDI has been proposed for high signal-to-noise ratio (SNR), high fidelity, and efficient complex image processing and offers unique features including (1) complex signal processing with Gaussian noise behavior; (2) intrinsic removal of irrelevant signal weighting factors (e.g., coil sensitivities); (3) simplified processing procedure (no need for explicit coil combination) with very high degree-of-freedom; and (4) very high computational efficiency [42] Compared to previous brain multiparametric quantitative MRI methods, the MULTIPLEX showed advantages in the improvement of SNR and flexibility [14, 15] Recently, artificial intelligence-assisted image reconstruction, particularly deep learning algorithms, has catalyzed a paradigm shift in medical imaging by slashing MRI acquisition time [43–47] These algorithms exploit the inherent structure and patterns in the data, yielding reconstructions that outperform traditional methods in image quality. Enhanced visual fidelity brings anatomical details and pathological changes into sharper focus, thereby bolstering diagnosis accuracy and treatment planning. Besides, deep learning-based reconstruction excels in noise and artifacts reduction, a notable advantage in MRI where the SNR often suffers due to complex anatomy and air-filler cavities of the brain. The next version of the MULTIPLEX will use artificial intelligence-assisted image reconstruction to shorten the scan time and improve the SNR of MR images.

There existed several limitations in our study. A larger sample size with diverse demographic information is needed to confirm our results. We carefully recruited a cohort of PD patients with clinically homogeneous character in a relatively small sample size, which showed power analysis results larger than 0.70, which is a bit lower than 0.8 but was way much larger than 0.5 and could be reliable, thus we believe our results are worth reporting. While our quantitative parameter findings could potentially contribute to the diagnosis of Parkinson’s disease, it is imperative to validate the robustness of our results. The efficiency of this diagnostic model requires further development and confirmation through validation with an independent cohort with more diverse demographic information. Additionally, the clinical assessments of PD subjects were evaluated at their “on” state, thus our results may be influenced by the medication. We admitted that clinical assessments at “off” state may more accurately describe the natural pathological changes of PD. However, the “on” state assessment reflects the real-world scenario where PD patients often take medication to manage their symptoms. When patients’ symptoms are alleviated, it would enhance feasibility and patient cooperation during imaging obtaining. The “on-off” fluctuation for the clinical assessment may also affect the results. Moreover, assessing patients at “on” state minimizes the discomfort and distress under the unmedicated states, which follows the ethical considerations for patient well-being during research participation. How to balance patient comfort and accurately assess pathological changes in PD is still challenging. In future studies, we plan to apply a broader range of disease diversity (different disease stages, on and off state of medication, the dose of medications and the time interval between scans after taking medication, with and without cognitive impairment, etc.) in PD and establish a longitudinal cohort to assess possible brain microstructural alterations derived from the MULTIPLEX sequence throughout the disease progression, as well as to evaluate prognosis after therapy. Moreover, as mentioned above artificial intelligence-assisted image reconstruction is applied in the next version of MULTIPLEX technique for shorter scan time and SNR improvement. The utility of MULTIPLEX as a multi-parametric MR imaging method still requires clinical validation in the clinically routine MR examination of PD patients.

## Conclusions

MULTIPLEX could provide a series of comprehensive and quantitative parameters that may relate to microstructural changes in the subcortical nucleus and dopaminergic midbrain regions in early PD patients. With the advantage of time-saving, it is a promising MR sequence to apply in clinics to prevent patients from unbearably long time MR examinations. Thus, it might be feasible for clinical application to assist in PD diagnosis and may provide possible pathological information of PD patients’ subcortical nucleus and dopaminergic midbrain regions.

## Data Availability

The datasets used and analysed during the current study are available from the corresponding author on reasonable request.

## Abbreviations

AUC: area under the curve
BOLD: Blood-oxygenation-level-dependent
CN: caudate nucleus
DBS: deep brain stimulation
DTI: diffusion tensor imaging
FDR: false discovery rate
Gpe: external globus pallidus
GPi: internal globus pallidus
H&Y: Hoehn and Yahr
HCs: healthy controls
HN: habenular nuclei
MDI: multi-dimensional integration
MDS: Movement Disorder Society
NAC: nucleus accumbens
PD: Parkinson’s disease
Pu: putamen
qMRI: quantitative MRI
QSM: quantitative susceptibility mapping
RN: red nucleus
ROC: receiver operating characteristic
ROIs: regions of interest
SNc: pars compacta of substantia nigra
SNpc: substantia nigra pars compacta
SNR: signal-to-noise ratio
SNr: pars reticulata of substantia nigra
SNR: signal-to-noise ratio
STN: subthalamic nucleus
T2*: T2 star
TE: echo time
Thal: thalamus
TR: repetition time
UPDRS: Unified Parkinson’s Disease Rating Scale
VeP: ventral pallidum
